# Intraspecies Variation in *Tetrahymena rostrata*

**DOI:** 10.3390/microorganisms9102100

**Published:** 2021-10-05

**Authors:** Anne Watt, Neil Young, Ruth Haites, Kerry Dunse, Derek Russell, Helen Billman-Jacobe

**Affiliations:** 1 Asia-Pacific Centre for Animal Health, Melbourne Veterinary School, Faculty of Veterinary and Agricultural Sciences, University of Melbourne, Parkville, VIC 3010, Australia; watta@unimelb.edu.au (A.W.); ruth.haites@unimelb.edu.au (R.H.); derek.russell@unimelb.edu.au (D.R.); 2 Melbourne Veterinary School, Faculty of Veterinary and Agricultural Sciences, University of Melbourne, Parkville, VIC 3010, Australia; nyoung@unimelb.edu.au (N.Y.); kmdunse@gmail.com (K.D.)

**Keywords:** ciliophora, mtSSUrRNA, histone, cytochrome oxidase 1, origin of replication

## Abstract

Two distinct isolates of the facultative parasite, *Tetrahymena rostrata* were compared, identifying and utilising markers that are useful for studying clonal variation within the species were identified and utilised. The sequences of mitochondrial genomes and several nuclear genes were determined using Illumina short read sequencing. The two *T. rostrata* isolates had similar morphology. The linear mitogenomes had the gene content and organisation typical of the *Tetrahymena* genus, comprising 8 tRNA genes, 6 ribosomal RNA genes and 45 protein coding sequences (CDS), twenty-two of which had known function. The two isolates had nucleotide identity within common nuclear markers encoded within the histone H3 and H4 and small subunit ribosomal RNA genes and differed by only 2–4 nucleotides in a region of the characterised actin genes. Variation was observed in several mitochondrial genes and was used to determine intraspecies variation and may reflect the natural history of *T. rostrata* from different hosts or the geographic origins of the isolates.

## 1. Introduction

*Tetrahymena rostrata* (Kahl 1926, Corliss) are ciliated protozoa which can be free-living in edaphic environments [1,2,3,4,5] and can occur as facultative parasites of terrestrial molluscs [2,6,7,8]. Most reports of natural infections of slugs and snails have shown that *T. rostrata* favours the renal tissues where it can multiply up to large numbers [8,9]. Histological examination of wild slugs has shown that the albumen gland and genital tract organs can become heavily infected with *T. rostrata* which can result in trans-ovarial transmission of the parasite [9]. There are few isolates of *T. rostrata* available for genetic comparison. Characterisation of isolates from *Helix aspera* snails and *Deroceras reticulatum* slugs in Spain included morphometric measurements and molecular data derived from the mitochondrial cytochrome oxidase 1 (*cox*1) gene and the nuclear small subunit ribosomal RNA gene (SSUrRNA) [10]. A further four isolates from *Zonitoides nitidus* and *Cochlicopa lubrica* snails from Poland were compared to the Spanish isolates using partial *cox*1 and SSUrRNA sequences. These comparisons showed the Spanish and Polish isolates were identical in the SSUrRNA but clustered as two subgroups based on *cox*1 [6]. The first group, consisting of isolates TR01, TR02, TR03, TR1034 and TR1035, had 0.9–1.3% sequence divergence. The second group, consisting of TR1015 and TR1016 had 0.6% divergence between them but a 4.4–5.2% sequence divergence from TR01, the type strain.. The guideline for declaring that *Tetrahymena* strains belong to different species is 4–5% divergence in the *cox*1 gene sequences [11,12]. Kaczanowski et al. suggested that the natural environment of *T. rostrata* is variable and that there may be a corresponding clonal diversity within the species [6].

We are interested in this species because of its potential as a biological agent for the control of pest slugs. A clear understanding of the variations within the species and development of molecular tools to study populations will inform decisions about release of the agent. Characterisation of the mitochondrial genomes of the two isolates gives us some insights into the conserved and variable genes and helps to identify targets for developing tools for molecular identification of isolates and closely related *Tetrahymena* species. In a broader context, this species is often overlooked in larger taxonomic studies of *Tetrahymena*, probably due to the scarcity of sequence data. We hope this work will encourage others to obtains sequences and to report on *T. rostrata* they may isolate, so that we can build a greater understanding of the population structure.

The Grey field slug, *D. reticulatum* is an invasive pest in many countries, including Australia where it has been present since at least the 1930s, probably longer [13]. *T. rostrata* was recently identified in Australia when it was isolated from the egg of a *D. reticulatum* slug and has been established in axenic culture [14]. The isolate, designated TRAUS, had the same morphological and phenotypic characteristics as the Spanish and Polish isolates. We recently reported the mitochondrial genome of TRAUS from Illumina libraries of whole cell DNA extracts [14]. While the TRAUS isolate grouped with Polish isolates TR1015 and TR1016 on the basis of the *cox*1 sequence data, the lack of whole mitochondrial genome sequence data for other *T. rostrata* isolates made detailed comparisons of taxonomic relationships challenging.

Phylogenetic studies of ciliates are increasingly being performed using additional nuclear and mitochondrial molecular markers, such as the mitochondrial SSUrRNA (mtSSUrRNA) and the nuclear ITS1-5.8S-ITS2 [12,15]. In this paper, we report the mitochondrial genome and selected nuclear genes of the type strain TR01 (an isolate from Spain), and make a comparison with our Australian isolate (TRAUS) to gain insights into variation within the *T. rostrata* species. We chose these isolates because of their widely separated collection sites.

## 2. Materials and Methods

### 2.1. Strains and Culture

*T. rostrata* TR01 was obtained from the American Type Culture Collection (ATCC^®^PRA326™). *T. rostrata* TRAUS was isolated from the egg of a laboratory-reared *D. reticulatum* in 2015. The parents of the slug had been previously collected from Ringwood North, SE Melbourne, Australia. The isolates were routinely cultured at 20 °C in sterile PPYE medium consisting of 0.5% *w/v* proteose peptone (Oxoid LP0085), 0.5% *w/v* yeast extract (Oxoid LP0021), and 0.125% *w/v* glucose. The source of the isolates and relevant sequences are listed in Table 1.

### 2.2. Morphological and Phenotypic Characterisation

*T. rostrata* TRAUS cells from a densely growing PPYE culture (cell density: 0.7–1 × 10^5^ cell/mL), were subcultured in PP (1% *w/v* proteose peptone (Oxoid LP0085) and 0.125% *w/v* glucose) for 7 days and were harvested (800× *g* 5–10 min) and washed twice in 10 mM HEPES pH7 NaOH. Cells were resuspended in an aqueous infusion of composted pine bark/10 mm HEPES pH7 NaOH and then dispensed into 6 well tissue culture plates. Plates were incubated at 26 °C for 24 h to allow cysts to form. Light microscopy was used to confirm that cells with rounded morphologies were cysts. Excystment was stimulated by transferring the trays to 20 °C and the released theronts were examined. Wet mounts and fixed Giemsa stained cells were imaged with a Leica DMLS light microscope. Cell measurements were made using ImageJ using a calibrated scale [16].

For scanning electron microscopy, trophonts were collected by centrifugation, washed and then fixed with 2.5% glutaraldehyde and applied to polyethyleneimine coated glass coverslips. Cells were dehydrated in increasing concentrations of ethanol and the coverslips were dried in a Balzers CPD030 critical point dryer (Balzers, Liechtenstein, Germany) and mounted onto 25 mm aluminium stubs with double-sided carbon tabs. The coverslips were coated with gold using a Xenosput sputter coater (Dynavac, Wantirna South, Australia). The cells were imaged with the Philips XL30 field-emission scanning electron microscope (Philips, Eindhoven, The Netherlands) at a voltage of 2.0 kV and a spot size of 2. Line art was made in Adobe Photoshop.

### 2.3. Genome Sequencing

Genomic DNA was extracted from axenic cultures of TR01 (ATCC PRA326) and TRAUS using a Promega genomic DNA kit. The DNA was fragmented by sonication and fragments in the suitable size range were purified and end-repaired and A-tailed using the polymerase activity of Klenow fragment. Indexed adapters were ligated to the DNA fragments by DNA ligase followed by performing a PCR reaction of 15 cycles to enrich the adapter-modified DNA fragments using KAPA HiFi HotStart ReadyMix (KK2602 Kapa Biosystems). After validating the libraries by TapeStation, each library was sequenced using Illumina MiSeq. The reads were mapped to the mitochondrial genome of the closest *cox*1 relative for which there was sequence in Genbank, *T. pigmentosa* (DQ927305) and then the mapped reads were self-assembled and used as scaffolds for rounds of gap filling and extension, using the total read libraries until the whole mitochondrial genome was assembled as a single contig. TRAUS and TR01 mitochondrial DNA sequences are available in GenBank (MN025427 and MT375014 respectively). Illumina read data is available from the GenBank Sequence Read Archive for TR01 SRR12315381 and TRAUS SRR12315411. The contigs were annotated with reference to the mitogenomes of *T. thermophila* (AF396436) [17], *T. pyriformis* (AF160864) [18], *T. paravorax* (DQ927304), *T. malaccensis* (DQ927303) and *T. pigmentosa* (DQ927305) [19].

### 2.4. Phylogenetic Analysis

The *cox*1 sequences for *T. rostrata* strains were obtained from Genbank and a 1796 nt *cox*1 barcode region was selected based on previous studies [11,20]. The mitochondrial SSUrRNA barcode region selected was the 541 bp identified by Doerder [12]. Alignments of *cox*1, and mtSSUrRNA were performed using MAFFT version 7.388 [21] and Bayesian phylogenetic inference was performed using a Markov chain Monte Carlo (MCMC) analysis in MrBayes version 3.2.6 (https://github.com/NBISweden/MrBayes, accessed on 1 June 2020) using a 1,100,000 MCMC generation chain length with consensus trees generated using the 50% majority rule criterion and the final 90% of trees generated by Bayesian inference after a burn-in of 100,000 generations. Estimates of evolutionary divergence between the 1796 nt *cox*1 and 689 nt *cox*1 barcode [20] were conducted using the Kimura 2-parameter model [22]. All ambiguous positions were removed for each sequence pair (pairwise deletion option). Evolutionary analyses were conducted in MEGA X [21] using the settings for protozoal mitochondria codon usage. Each functional pair of coding DNA sequences was extracted from the assembled TR01 and TRAUS mtDNA contigs and aligned in Geneious Prime using the Translate align function with the mold-protozoan mitochondrial genetic code table and Blosum 62 cost matrix [23]. Single stranded DNA topology was determined using DNAfold in Geneious Prime.

## 3. Results and Discussion

### 3.1. Morphological and Phenotypic Examination of Isolate TRAUS

**Figure 1:**
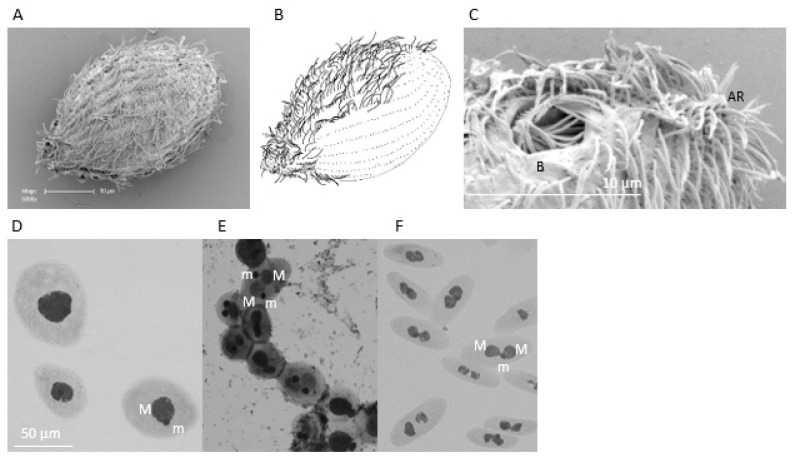
(**A**) Scanning electron micrograph and (**B**) line drawing of *T. rostrata* TRAUS showing a trophont with 28 rows of cilia and a tapered rostrum; (**C**) scanning electron micrograph of the buccal opening (B) and anterior rostrum (AR); Giemsa stained (**D**) trophonts, (**E**) cysts and (**F**) theronts showing macronuclei (M) and micronuclei (m).

The *Tetrahymena* isolate, designated TRAUS, showed the typical morphology of *T. rostrata* and was capable of forming cysts and releasing theronts, as originally shown for *T. rostrata* by Corliss [1]. These data show that TRAUS has the same morphological and life history characteristics as other isolates of *T. rostrata* described by other authors [9,10]. Trophonts were ovoid and may have a rostrate anterior end (Figure 1A). Fresh, unfixed trophonts grown in PPYE for 7 days were an average size of 56.45 ± 7.91 × 42.08 ± 6.69 µm (*n* = 78). The oval buccal opening was situated in the top quarter of the anterior end and was lined with ciliary membranelles (Figure 1C). Trophonts contained a macronucleus and associated micronucleus (Figure 1D). Trophonts developed into cysts under nutrient deprivation at 26 °C and the cysts released theronts with characteristic lobed macronuclear analgen either side of a micronucleus (Figure 1D–F). The trophonts had ~28 kineties and the oral opening was ~11 × 9 microns.

### 3.2. Mitogenomes of TR01 and TRAUS

The mitogenomes of TRAUS and TR01 were characterised using 17,797,914 and 33,260,090 Illumina DNA short-read sequences, respectively [14]. Assembly of the mitochondrial DNA sequences of TRAUS resulted in a 47,235 nt contig (1,498,614 reads, average sequence depth of 4806 reads) and for TR01 a 47,310 nt contig (1,204,576 reads, average sequence depth of 3808 reads). The mitogenomes were linear and the assemblies reached the telomeric repeats which was taken as an indication of assembly of the compete mitochondrial genomes.

The TRAUS mitogenome had a percent nucleotide composition of T (40.6) C (10.5), A (37.6) and G (11.2) which was almost identical to the TR01 mitogenome composition which was T (40.6), C (10.6), A (37.7) and G (11.1). Both had a low G + C content (21.7–21.8%). Each had 45 protein coding sequences (CDS), 8 tRNA genes and 56 ribosomal RNA genes. There are 2 ORFS for the SSUrRNA (rns a and rns b) and two ORFS for the LSU rRNA rnla and b. The rnl a and b genes are duplicated at each end of the linear mitochondrial DNA. Twenty-two of the coding DNA sequences (CDS) encoded proteins of known function and the remaining CDS were open reading frames designated *ymf*, which are conserved in *Tetrahymena* but have no known function (Table 2) [17,18,19]. The difference in the length of the contigs of TR01 and TRAUS, excluding the telomer repeats, consist of 24 InDels in intergenic regions and InDels in rnlb1 (4 SNPs), rnsb (2 SNPs) and rnlb2 (1 SNP).

**Figure 2:**
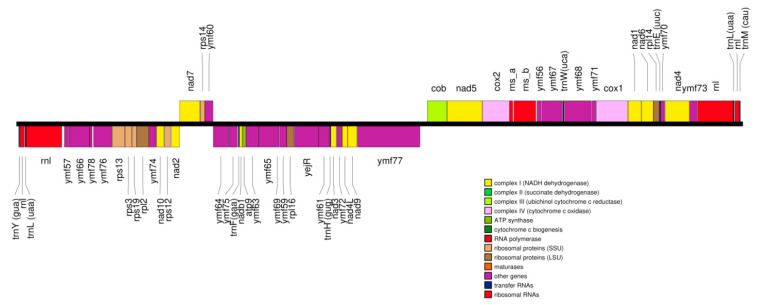
Arrangement of ORFS in the *T. rostrata* mitogenome.

**Figure 3:**
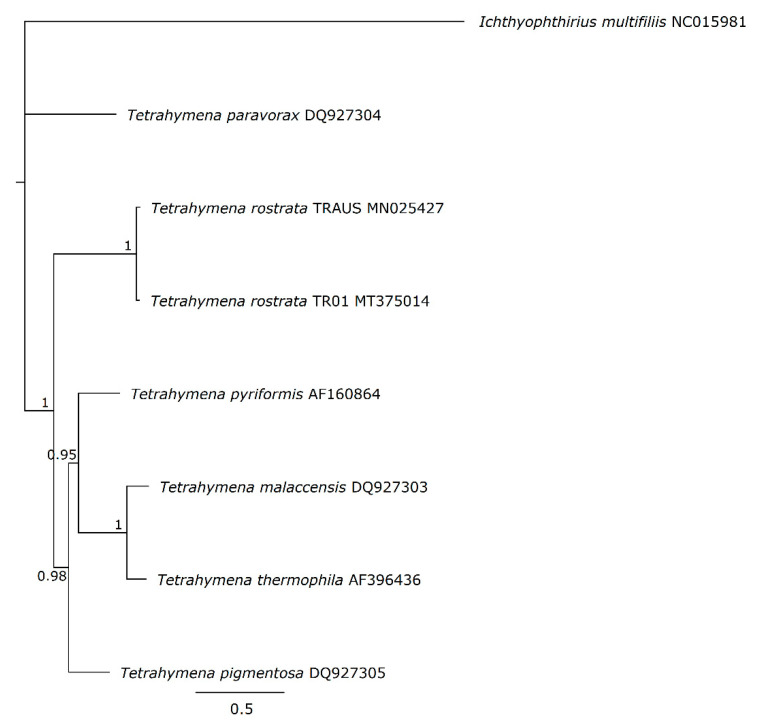
MrBayes tree of the translated, concatenated amino acid coding regions of the mt genomes of *T. rostrata* TRAUS, TR01 and other *Tetrahymena* species. The MrBayes tree was built in Geneious Prime v.1.2 using a 1,100,000-generation chain length and a 1,000,000-generation burn-in with *Ichthyophthirius multifiliis* as an out group. Posterior probability values are shown to indicate branch support.

The organisation and gene arrangements are syntenic with the other *Tetrahymena* mitogenomes; *T. thermophila* [17], *T. pyriformis* [18], *T. paravorax, T. malaccensis* and *T. pigmentosa* [19] except that there was no duplication of *nad*9, as has occurred in *T. thermophila* and *T. malaccensis*. The genes are arranged divergently from a central region which is thought to contain elements that control transcription and replication (Figure 2). Phylogenetic placement of the concatenated amino acid coding regions of the mitogenomes placed TRAUS and TR01 closely together (Figure 3).

Twenty-two ORFs encoding proteins of unknown functions were identified in the *T. pyriformis* genome and were designated *ymf*56 to 77 [18]. Three were subsequently assigned functions [17] and an additional gene, *ymf*78 was identified after proteomic analysis of *T. thermophila*. At least 13 of the *ymf* genes are expressed in *T. thermophila*, including *ymf*78 [5]. The occurrence and arrangement of the *Tetrahymena ymf* genes are conserved in *T. rostrata*. Ymf78 peptide, which is highly conserved within the genus, was identical in the two *T. rostrata* mtDNA sequences studied.

### 3.3. Cytochrome Oxidase 1 Gene

**Figure 4:**
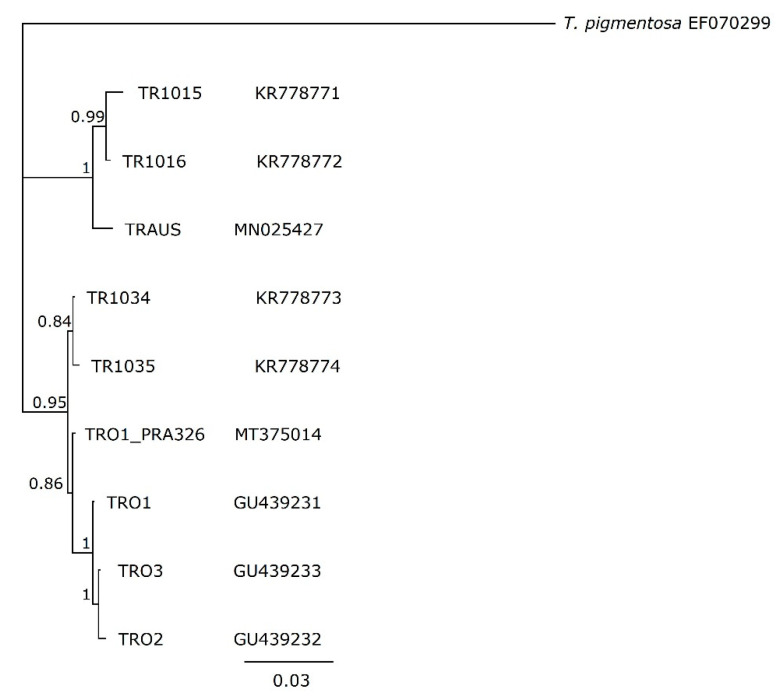
Phylogenetic tree of *Tetrahymena rostrata* strains based on 1796 bp of the *cox*1 genes. A MrBayes tree was built in Geneious Prime v.1.2 using *Tetrahymena pigmentosa* as an outgroup. The MrBayes tree was built in Geneious Prime v.1.2 using a 1,100,000-generation chain length and a 1,000,000-generation burn-in with *Tetrahymena pigmentosa* as an outgroup. Posterior probability values are shown to indicate branch support.

Comparison of the available reported sequences of 1796 nucleotides for the *cox*1 gene of *T. rostrata* isolates is shown in Figure 4. The phylogenetic analysis shows that the sequences group into two clades. Sequences of TRAUS, TR1015 and TR1016 cluster together. The genetic distances between TRAUS and TR1015 and TR1016 were 1.52 and 1.07 respectively. The sequences of the other Spanish and Polish isolates clustered together as previously reported [6]. There was no discrimination dependant on the host animal or geographic origin of the isolates. The *cox*1 sequence previously deposited in Genbank for TR01 is included (GU439231). There were 13 SNP differences between the *cox*1 of TR01 entered into Genbank in 2016 [11] and TR01 (ATCCPRA326) (MT375014) as examined by us. The Illumina reads across the TR01 (ATCCPRA326) *cox*1 gene are homogeneous indicating that the template is clonal. The most likely reason for the discrepancy is that the TRO1 *cox1* GU439231 was derived from DNA extracted from a *T. rostrata* culture that was not clonal, as indicated in Segade et al., 2009 [10], but the material deposited at ATCC is clonal. The *cox*1 sequence of TR01 (GU439231) and TR01_PRA326 (MT375014) agree over the first 1064 bases of common sequence. Indeed, TR02 and TR03 sequences are also the same over this region which covers the barcode region (nt 220-908, MT375014). The 18S sequence did not differ between TR01 (JQ 045342), TR01 (ATCCPRA326) and TRAUS. Comparison of each protein coding DNA sequence showed that there was a high level of conservation across the mitogenomes. There were no particular hotspots of nucleotide substitutions or indels that might indicate genetic drift.

### 3.4. HCEs and Central Repeat Region

**Figure 5:**
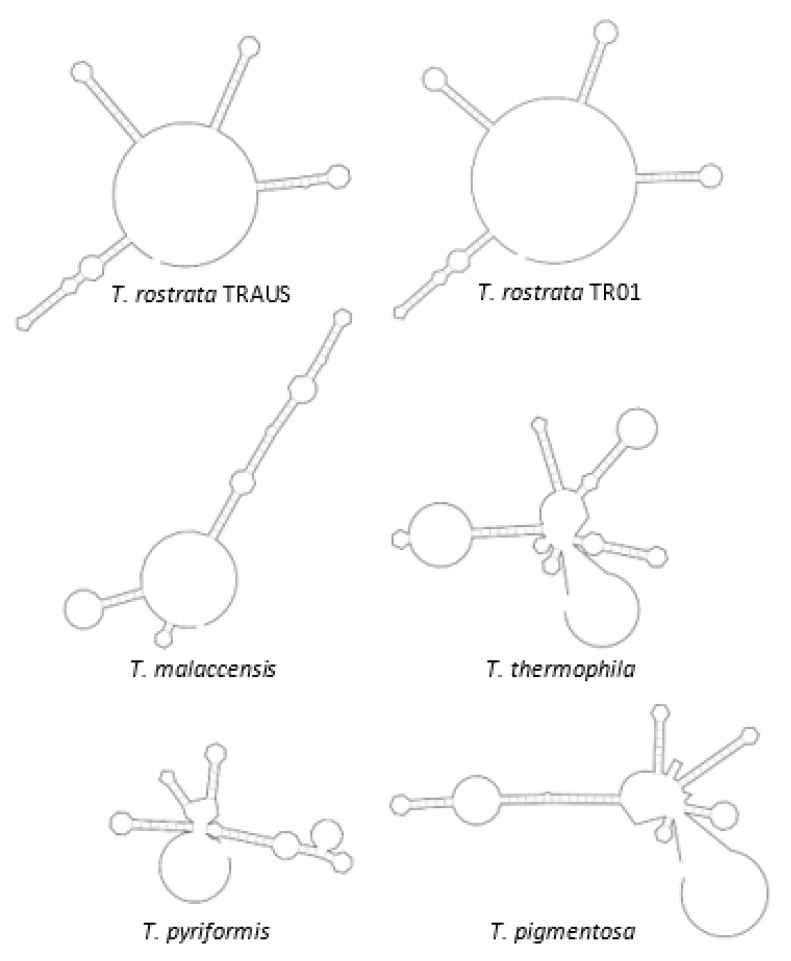
Secondary structures of the central repeat region of *T. rostrata* TR01 and TRAUS and other *Tetrahymena* species based on single stranded DNA.

Five highly conserved elements (HCE) have been identified in the mitogenomes of other *Tetrahymena* [19,24]. The HCEs occur in both TR01 and TRAUS at the same sites. HCE287 was 31 nt downstream of *ymf*57. Both HCE234 and 290 are overlapping and occur within the coding region of *ymf*78. HCE299 occurs in the *nad*2 CDS and has been suggested to be the promoter of *nad*7 [24]. HCE315 and HCE138 occur between the *ymf*77 and *cob* ORFs which are arranged divergently, on either side of the central control region. Notably HCE138, 29 bp (5′AATAGCCGCACCAAAAAGAAAAAAATCAA) was shared with the other species of *Tetrahymena* (Table 3). The motif contains the very highly conserved, GC-rich motif, GCCGCACC [19]. The only pair to agree completely were TRAUS and TR01 The distance between HCE138 and HCE315 was 113 nt for TRAUS and 120 nt for TR01. Each isolate had 5 tandem repeats between HCE138 and HCE315, but the repeats differed The TRAUS repeat was TAAATTTAAAATAAT and the TR01 repeat TAATTAATTAAAAATAA. Repeats were not apparent in the other species. The region between HCE138 and 315 contains the most variable spans of sequence between TR01 and TRAUS. However secondary structures were almost identical and were significantly different from that of the other species (Figure 5). Zhang et al. [25] compared the central region of a number of linear ciliate mitogenomes and found the repeats in two species of *Euplotes* were identical. However, comparison shows that the translated *nad*9 genes in these two species are only 63.92% similar highlighting how multiple sequences are needed to build taxonomies.

### 3.5. mtSSUrRNA

**Figure 6:**
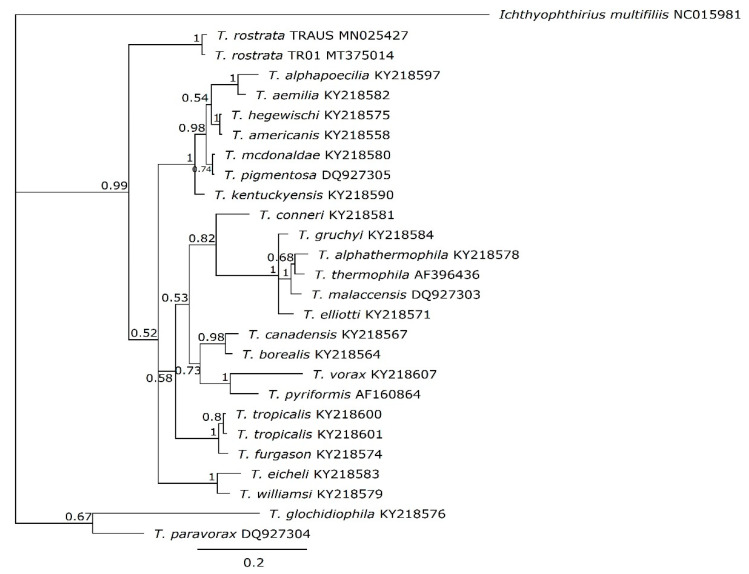
Phylogenetic tree of *Tetrahymena* based on the mtSSUrRNA, 541 bp right-hand region of TRAUS, TR01 and other *Tetrahymena* species. The MrBayes tree was built in Geneious Prime v.1.2 using a 1,100,000-generation chain length and a 1,000,000-generation burn-in with *Ichthyophthirius multifiliis* as an out group. Posterior probability values are shown to indicate branch support.

Doerder [12] highlighted the use of the mtSSUrRNA for species identification where there are multiple isolates available. Doerder found that the “left” and “right” region of the mtSSUrRNA were highly variable and that the right region was represented by more strains in GenBank. The right mtSSUrRNA region was analysed for its potential ability to provide clarity around complex species. The two *T. rostrata* mtSSUrRNA have 2 SNP differences (1 transition and 1 transversion) indicating a low sequence divergence (2.7%). Both isolates had identical V4 regions (TRAUS nt847-1008; TR01 nt848-1009). A phylogenetic tree of the mtSSUrRNA sequences shows TR01 and TRAUS on a separate branch from other *Tetrahymena* (Figure 6). Intra-species variability has been found in some other *Tetrahymena* species [12]. In this case, we are able to compare the whole mtSSU genes of the two isolates. Overall, there are 18 SNP differences in the 1431 nt gene and 8 of the SNPs are focused in a 22–25 nt hypervariable domain. The differences are one 3 nt insertion (TTT), 3 transversions and 2 transitions. This region (TR01 nt1283-1304) may be particularly useful for examining diversity in species complexes. It is situated 3′ to the region most commonly available in Genbank which was used in recent *Tetrahymena* phylogenies which revealed many new species [12]

### 3.6. Nuclear Genes

Nuclear genes are expected to evolve at a different rate from mitochondrial genes. However, the life cycle of *T. rostrata* is different from ciliates which have genetic exchange via conjugation. During encystment-induced autogamy, the pronuclei from the same cell fuse and the new macronuclei derived from them are expected to be homozygous [6,26]. Recessive mutations in the micronuclei will accumulate though successive rounds of autogamy unless there is purifying selection. We mined the short read files to extract several nuclear genes. The entire small subunit, 18S rRNA-ITS1-5.8S-ITS2-28S rRNA sequences of TR01 and TRAUS were assembled as ~6 kb single contigs for each strain (MT420428 and MN158348 respectively). Contigs were assembled with 168,388 reads for TR01 and 656,235 reads for TRAUS with an average coverage of 4299 for TR01 and 16,024 for TRAUS. These were compared to Genbank entries for 18S rRNA available for a *T. rostrata* from Spain (JQ045342) (strain not specified) and the Polish strains, TR1015, TR1016 TR1034 and TR1035. The entire set are identical across the 18S-ITS1-5.8S rRNA sequence and 28S sequence. Neither *T. rostrata* TR01 nor TRAUS have a Group 1 intron in the 28S rRNA [27].

Histone H3—intergenic region—histone H4 sequences of TR01 and TRAUS were each assembled as single contigs for each strain (MT506240 and MN167836 respectively). The entire H3 and H4 genes were covered. Contigs were assembled with 259 reads for TR01 and 345 reads for TRAUS with an average coverage of 20 reads for TR01 and 30 reads for TRAUS. The H3 and H4 histone genes of TR01 and TRAUS were identical, two SNPs were identified in the intergenic region.

Actin plays an essential role in multiple eukaryotic cellular process such as cytoskeletal structure, motility and intracellular transport. Many eukaryotes have families of actin genes. However *T. thermophila* and *T. pyriformis* have just one actin gene without exons. There are low levels of genetic polymorphism within the actin gene of different populations of *T. thermophila* [28] but other ciliates have variation within and between actin gene families [29]. We attempted to extract the actin gene sequence from TRAUS and TR01 for comparison, using the actin gene of *T. thermophila* template. Surprisingly, 4 contigs were assembled from each short read library, which were labelled ACT1-4. Each encoded a 377 aa protein which had 99.73% similarity to *T. thermophila* actin. The paralogs for each pair had low sequence divergence between TRAUS and TR01 (ACT1, 0; ACT2, 0.5%; ACT3, 0; ACT4, 0.4%). However, the divergence between alleles from TRAUS was between 4.2% and 7.3% and the equivalent comparison for TR01 paralogs was 4.4–7.2% divergence. These findings indicate that duplication of actin genes has occurred in *T. rostrata* and there must be multiple actin genes in the micronuclear genome which are destined for the macronucleus. The multiplication of the actin genes is most likely to have occurred in the last common ancestor of TRAUS and TR01 because the divergence between the pairs (e.g., the 2 ACT1 genes) is less than the divergence of the ACT genes within the strain. It appears that there have been three duplication events from an ancestral actin gene, first to produce two genes, which in turn were both duplicated. Between the ACT gene pairs in TRAUS and TR01, the copies of ACT4 and of ACT3 were identical and there were 4 and 2 SNPS between the ACT1 and ACT2 pairs, respectively, showing the between pair conservation is high and suggesting that, if all mutations were equally likely, that ACT4 and ACT3 were the more recent duplicates.

## 4. Conclusions

We have compared *T. rostrata* TRAUS with TR01 on the basis of morphology and life cycle plus by comparison of 58 DNA sequences (9 nuclear genes and 3 ITS, and 45 mitochondrial genes and the central repeat region). This is the most extensive comparison of any two *Tetrahymena* isolates to date. Classical mating experiments to assign them to the same species are not possible with *T. rostrata* because it does not form mating pairs and therefore we must rely on morphology, life cycle and molecular comparisons to determine the relationships between isolates. In every respect, TRAUS and TR01 are more like each other than with any other *Tetrahymena.* There was a high level of identity in nuclear genes although there is some diversity in the mitochondrial genes, which are expected to evolve at a higher rate. The variability in the protein coding mitochondrial CDS was similar among some *ymf* genes and genes encoding known proteins. Some mitochondrial genes, such as *nad*9, are particularly useful for comparison because we can compare the rate of change in the duplicates in *T. thermophila* and *T. malaccensis* and *cox*1 because it is represented by many sequences in the databases and is variable. The examination of difference in the *cox*1 barcode region shows the two isolates to be very closely related and difference are not sufficient to split them into separate species. None of the other comparisons suggested differences beyond intraspecies variation. Such differences as there are might be attributed to clonal variation between two isolates of *T. rostrata* from very distant geographical sites and possibly due to some selection during their parasitic phase in different hosts. The whole genome sequencing was a relatively easy way to derive the mitochondrial genomes and had the added bonus that we could mine the short read archive for additional nuclear genes. A complete genome assembly using long reads will further elucidate features of this species.

## Figures and Tables

**Table 1:** *Tetrahymena rostrata* isolates and sequences.

Strain ID	Host	Tissue	Accession Numbers for Each Isolate	Ref.
TR01	*Helix aspersa aspersa*	renal organ	GU439231 (*cox*1), JQ045342 (18S rRNA)	[9]
			MT375014 (mtDNA), MT506240 (histone H3 H4), MT420428 (18S-5.8S-28S rRNA), SRR12315381(short read archive)	This study
TR02	*Helix aspersa maxima*	renal organ	GU439232 (*cox*1)	[9]
TR03	*Deroceras reticulatum*	renal organ	GU439233 (*cox*1)	[9]
TR1015	*Zonitoides nitidus*	renal organ	KR778771 (*cox*1); KR778775 (18S rRNA)	[5]
TR1016	*Zonitoides nitidus*	renal organ	KR778772 (*cox*1), KR778776 (18S rRNA)	[5]
TR1034	*Cochlicopa lubrica*	renal organ	KR778773 (*cox*1), KR778777 (18S rRNA)	[5]
TR1035	*Cochlicopa lubrica*	renal organ	KR778774 (*cox*1), KR778778 (18S rRNA)	[5]
TRAUS	*Deroceras reticulatum*	egg	MN025427 (mtDNA), MN167836 (histone H3	
			H4), MN158348 (18S, 5.8S, 28S rRNA), SRR12315411 (short read archive)	[12] and this study

**Table 2:** Comparisons of protein coding sequences of TRAUS and TR01.

CDS	bp	DNA Seq Identity (%)	Amino Acid Seq Similarity (%)
Atp9	228	99.12	100
Rps14	306	99.02	100
Ymf57	303	98.68	100
Ymf78	168	98.21	100
Ymf56	294	97.96	100
Ymf72	357	97.76	99.16
Nad4L	351	97.44	100
RpS12	402	97.26	100
Ymf69	216	97.22	100
Ymf61	720	97.08	99.58
Ymf65	2652	97.05	100
Nad2	537	97.02	100
Ymf64	999	97	99.7
Nad10	489	96.93	100
Ymf76	1194	96.82	99.75
RpS3	456	96.77	100
RpL16	465	96.77	100
Ymf74	492	96.75	100
Cob	1287	96.74	99.77
Nad1b	180	96.67	100
Ymf70	270	96.67	98.89
Nad7	1329	96.61	99.77
RpS13	834	96.52	99.28
Nad3	366	96.45	100
Ymf75	567	96.3	98.41
Ymf63	816	96.2	100
Cox1	2067	96.18	99.71
RpL2	789	96.07	100
RpS19	297	95.96	98.99
Ymf66	1329	95.71	100
Nad1a	855	95.67	100
Ymf68	1761	95.57	99.32
RpL14	360	95.56	100
Nad4	1578	95.56	97.72
YejR	1581	95.51	98.67
Nad6	762	95.41	98.92
Ymf60	534	95.13	99.44
Ymf67	1344	95.09	98.44
Ymf73	483	95.03	100
Nad9	597	94.97	98.99
Nad5	2280	94.91	99.21
Cox2	588	94.9	98.64
Ymf59	471	94.69	98.73
Ymf71	264	94.32	97.73
Ymf77	4077	92.62	96.98

**Table 3:**
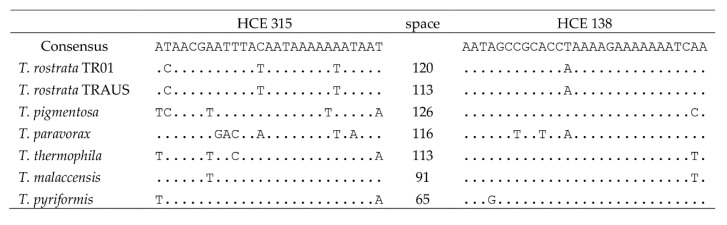
Comparison of HCE sequences in between *ymf77* and *cob* in *Tetrahymena* species.

## Data Availability

All sequences have been deposited in Genbank.
